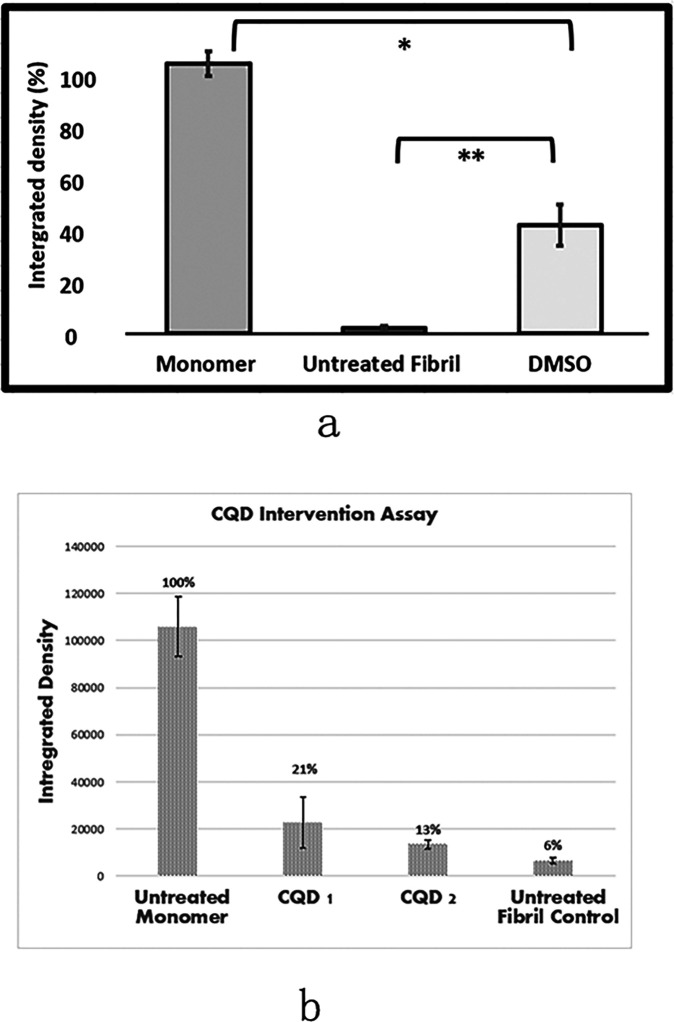# Screening Carbon Nano Materials for preventing amyloid protein aggregation by adopting a facile method

**DOI:** 10.21203/rs.3.rs-4164618/v1

**Published:** 2024-03-29

**Authors:** Daisy L. Wilson, Ana Carreon, Sampath Chinnam, Hamidreza Sharifan, Jyoti Ahlawat, Mahesh Narayan

**Affiliations:** The University of Texas at El Paso; the University of Texas at El Paso (UTEP); M.S. Ramaiah Institute of Technology (Autonoumous Institution, Affiliated to Visvesvaraya Technological University; the University of Texas at El Paso (UTEP); The University of Texas at El Paso; The University of Texas at El Paso

**Keywords:** amyloid proteins, soluble-to-toxic conversion, gel electrophoresis

## Abstract

The soluble-to-toxic transformation of intrinsically disordered amyloidogenic proteins such as amyloid beta (Aβ), α-synuclein, mutant Huntingtin Protein (mHTT) and islet amyloid polypeptide (IAPP) among others is associated with disorders such as Alzheimer’s disease (AD), Parkinson’s disease (PD), Huntington’s disease (HD) and Type 2 Diabetes (T2D), respectively. The dissolution of mature fibrils and toxic amyloidogenic intermediates including oligomers continues to be the pinnacle in the treatment of neurodegenerative disorders. Yet, methods to effectively, and quantitatively, report on the interconversion between amyloid monomers, oligomers and mature fibrils fall short. Here we describe a simplified method that implements the use of gel electrophoresis to address the transformation between soluble monomeric amyloid proteins and mature amyloid fibrils. The technique implements an optimized but well-known, simple, inexpensive and quantitative assessment previously used to assess the oligomerization of amyloid monomers and subsequent amyloid fibrils. This method facilitates the screening of small molecules that disintegrate oligomers and fibrils into monomers, dimers, and trimers and/or retain amyloid proteins in their monomeric forms. Most importantly, our optimized method diminishes existing barriers associated with existing (alternative) techniques to evaluate fibril formation and intervention.

## Introduction

A hallmark feature of neurodegenerative disorders such as AD, PD, HD and T2D is the soluble-to-toxic conversion of disease-associated prion-like amyloidogenic proteins such as Aβ, α-synuclein, mHTT, and IAPP, respectively (1–7). The formation of mature fibrils from their soluble, monomeric counterparts is often the “end-point” of the amyloid-forming (amyloidogenic) trajectory. Fibril formation is essentially deemed irreversible. Mature fibrils, which are rich in β-sheet content are insoluble and not easily amenable to structural studies.

Amyloid monomers are converted to mature fibrils via a sequential process that first results in the formation of from monomers, dimers and trimers to neurotoxic oligomers (7, 8). Oligomers are generally converted into proto-fibrils prior to the formation mature fibrils which is a terminal process as aforementioned. A comparison of the kinetics of monomer consumption relative the fibril formation is important. A difference in the rate of monomer consumption relative to fibril formation suggests the presence of intermediates, including kinetically-trapped aggregates (7). Quantifying the loss of monomers is essentially for a detailed biophysical understanding of the amyloidogenic trajectory. After all, it is the most experimentally tractable of all species along the amyloid-fibril-forming pathway. It informs us of whether the ambient conditions are biased towards retaining the monomeric conformation or towards fibril formation. It provides a mechanism by which to determine the rate at which monomers are consumed to form fibrils is also important. Measurement of the rate can then be used to fine-tune ambient conditions either to intervene in the fibrillation or to promote it (say, for biophysical studies) (9). Conversion of mature fibrils to their soluble monomeric counterparts is also indispensable for qualitative and quantitative evaluation of the efficacy by which small molecules may intervene (therapeutically or prophylactically) in amyloid-forming trajectories. Molecules such as tanshinone, brazilin and other aromatics along with specific carbon nano materials known as carbon quantum dots and graphene quantum dots have been instrumental in passivating amyloid monomers, remodeling oligomers, and dissolving mature fibrils (10–12). With respect to small molecule intervention, the ability to revert all non-monomeric intermediates including mature fibrils, to their soluble monomeric counterpart is key. Also critical is the ability to localize where along the fibril-forming trajectory that a small molecule intervenes is important for further advancing the candidacy of the said molecule.

Existing techniques to identify the presence of fibrils include dynamic light-scattering (DLS), microscopy (SEM, TEM, etc), x-ray fiber diffraction, solid-state NMR, and EPR among others (13, 14). While each technique offers specific advantages to the detection of fibrils, they also require equipment that is not easily accessible, is expensive, and/or requires extensive sample preparation. Furthermore, although the use of SDS page has been used previously used to assess the oligomerization of amyloid proteins; current methods rely on the use of crosslinking agents such as formaldehyde, or glutaraldehyde to form covalent bonds between adjacent subunits. (15–18) This practice is understandable when the aim is to analyze the structure of the protein, without the possibility of dissociation during electrophoresis. However, for the determination of efficacy of small molecule intervention, these additional steps are not necessary.

Here, we demonstrate a simplified method to use gel electrophoresis to determine the ability of small molecules to revert mature fibrils to their soluble monomeric counterparts (19). The advantages of this method over existing techniques is discussed.

## Method

### Gel Electrophoresis

12% Gels were prepared as described elsewhere (19, 20). Briefly, for the running buffer, 1650 uL of water, 2000 uL of 30% acrylamide, 1250 uL of 1.5 M Tris (pH 8.8), 50 uL 10% ammonium persulfate and 2ul TEMED was combined in a 15 mL falcon tube and transferred to the slides. Later, the layering was performed using tertiary butanol. The gels were then left to polymerize for about 20 minutes. The stacking solution containing 1550 uL of water, 250 uL of 30% acrylamide, 190 uL of 1.5 M Tris (pH 6.8), 15 uL ammonium persulfate and 1.5 ul TEMED in a 15 mL falcon tube was introduced into the gel on top of the running gel. The stacking gel was left for polymerizing for 15 minutes and then stored at −4 °C until further use in wet Kim wipes covered with the Aluminum foil.

### Preparation of Lysozyme solution

2 mg/mL of Lysozyme solution in freshly made potassium phosphate buffer (20 mM, pH = 6.3, 3M Guanidinium Hydrochloride) was prepared in a 5 mL glass vial. The glass vial containing lysozyme solution was then kept in an incubator-shaker at 550 rpm for 6 hours at 58 °C. After 6 hours, the contents of the glass vial were turbid which indicated the formation of lysozyme fibril (which was later confirmed using Transmission Electron Microscopy). Dialysis was performed to remove guanidinium hydrochloride (GdnHCl) salt as it compromises the quality of the stained protein bands (salt spread).

### Loading of amyloid samples onto the gel

The aforementioned dialyzed solution was added into the 1.5 mL Eppendorf tubes, which were then transferred to a tabletop ultracentrifuge. Centrifugation was carried out at 12,400 rpm for 15 minutes.

The supernatant was collected in 1.5 mL Eppendorf tubes and DI water was added to the pellet and mixed well. 30 uL of the solution (including supernatant and pellet) was then transferred in separate 0.5 mL pre-labelled Eppendorf tubes. Later, 10 uL of 4X loading dye was added to 30 uL of supernatant and pellet solution. Monomeric solution of Lysozyme (2mg/mL) was prepared as a control and 30 uL was mixed with 10 uL of 4X loading dye. The samples were then heated at 95 °C for 5 minutes and 20 uL of this solution was then loaded into the wells of the gel. The Gel was then run for 85 minutes at 120V and 400 A. For the staining-destaining procedure, the gels were removed from the glass slides and rinsed with water. Later, the gels were submerged in Coomasie staining solution overnight. The next day, destaining procedure was performed to destain the gels using 1:1 :0.2 ratio of water:methanol: acetic acid. The destaining was performed three times for 20 minutes each. After the third destaining wash, the gel was submerged in water to and an image was subsequently obtained using the Invitrogen iBright Imaging system.

### Data Analysis

The obtained images of the gel using the iBright imaging system were analyzed using the Image J software. The data obtained from Image J were transferred to Origin Pro software and mean and standard deviation values are calculated for each band. The bar graph is plotted against Integrated Density vs Sample name.

This simplified method using gel electrophoresis without crosslinking agents provides advantages over existing techniques, offering a cost-effective and accessible approach for assessing small molecule interventions in amyloidogenic trajectories.

## Results

**Figure 1:**
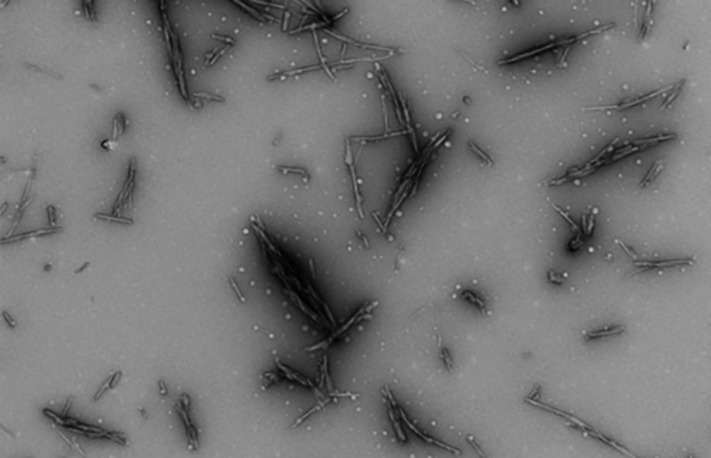
TEM image of fibrils

Figure 1 is a TEM image of mature HEWL fibrils. The fibrils are needle-form and well-delineated in nature. The mature fibrils appear to be interspersed with smaller, potentially, proto-fibrillar aggregates. The data are in good agreement with previous literature.

**Figure 2:**
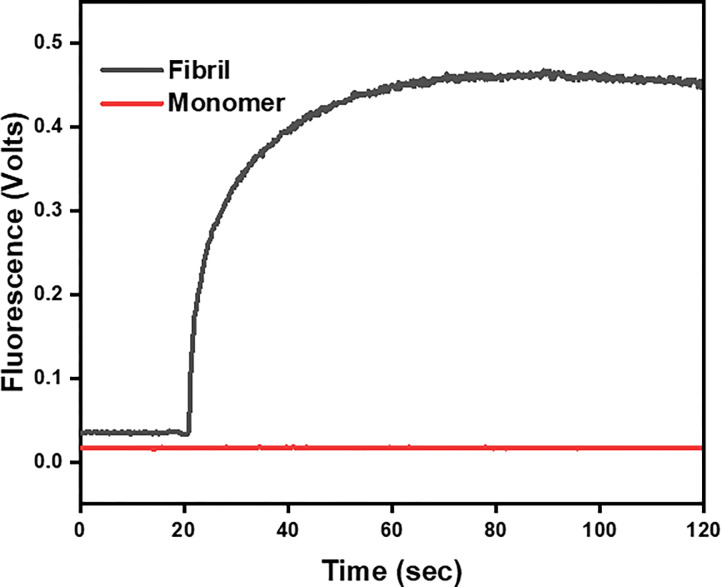
ThT assay confirming the presence of fibrils and monomers. Red: ThT added to a solution of lysozyme monomers. Black: ThT added to a solution containing mature HEWL fibrils.

Figure 2 shows the results of the ThT assay in which ThT was added (@20s) to a solution containing mature fibrils (black curve). There is a sharp and rapid increase in fluorescence upon introduction of the fluorophore, which is indicative of binding to the grooves in the cross-β structure of the fibrils. The plateauing of the curve suggests that all fibril is ThT bound or that there is no more free ThT. By contrast, the introduction of ThT to monomeric lysozyme (red curve) did not elicit any increase in fluorescence as is anticipated.

**Figure 3:**
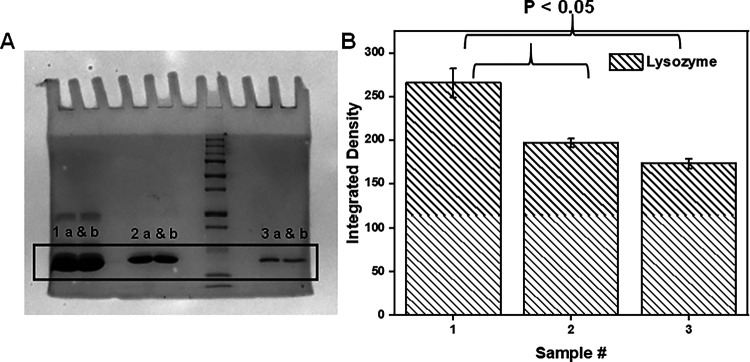
PAGE showing protein bands where 1a-b is monomers (starting protein solution; 2mg/mL), 2a-b is supernatant (remaining monomers after fibrillation) and 3a-b is pellet (fibrils). The data is plotted for N = 2 where p < 0.05 was observed.

Next, we determined whether gel electrophoresis could be used to qualitatively discriminate between HEWL mature amyloid fibrils and its monomeric counterpart. Figure 3 is an image of a PAGE experiment where HEWL monomers (3: 1 a and b), the supernatant (3: 2 a and b) from a centrifuged solution containing mature HEWL fibrils and a resuspended (3: 3 a and b) HEWL fibril pellet were loaded onto the gel. The location of the bands correspond to the molecular weight of monomeric HEWL. Inspection of the band intensities reveals that compared to the sample exclusively containing HEWL monomers (3: 1 a and b), there is a decrease in the intensity of HEWL monomers in sample (3: 2 a and b) and a further attrition in its concentration when sampled from the fibril pellet (3: 3 a and b).

Figure 3B shows quantified results from the aforementioned experiment. establish the presence of the presence of monomers and there consumption to form fibrils. Statistical significance was found across samples 3:1 and 3:2 and samples 3:1 and 3:3 indicating that PAGE can be used to quantify the soluble-to-fibril transformation of amyloid-fibril-forming proteins.

We tested whether small molecules and nanocarbon materials are able to revert HEWL fibrils to their soluble monomeric counterparts. Figure 4A shows the results from an experiment where HEWL fibrils were incubated with DMSO. There is an increase in the concentration of HEWL monomer when mature HEWL fibrils are exposed to DMSO, relative to untreated fibrils. Furthermore, the difference in monomeric HEWL concentrations between DMSO-treated fibrils and untreated fibrils is statistically significant. The data indicate that the DMSO-driven reconversion of mature fibrils to their monomeric counterpart can not only be easily visualized by our method but also quantified. The (statistically significant) difference in monomeric HEWL concentration between the monomer control and the DMSO-treated fibrils is also notable. In this scenario, the fraction of monomer released from DMSO-treated mature fibrils is reflects the small-molecule-driven fibril-to-soluble reconversion efficiency at the concentration of small-molecule used. Here too, a dose-response curve (obtained by varying the v/v of DMSO or the concentration of other small-molecules) can be constructed using this method.

Figure 4 B shows results obtained after exposing HEWL fibrils to carbon quantum dots (CQD1: citric; CQD2:gelatinized carbon). Although there appears to be a CQD-dependent increase in soluble monomers relative to the untreated fibrils, the results were not found to be statistically significant at the CQD dose employed.

## Discussion

The soluble-to-toxic conversion of amyloid proteins such as Aβ, α-synuclein, mHTT among others is a critical milestone in the onset and pathogenesis of amyloid-specific neurodegeneraive disorders. Efforts to address an understanding of this biophysical transformation are driven by spectroscopic and immunohistochemical tools. Nevertheless, access to instruments such as solid-state NMR, microscopes (TEM, HR-TEM, SEM, AFM), ATR-IR, DLS instruments and biochemical kits precludes routine studies of the process for many laboratories and investigators.

Even if high-resolution microscopes are accessible, extensive sample preparation protocols, analyses times and availability of very specific technical/instrumentation expertise are some of the criteria that remain as hurdles for researchers. Finally, and critically, microscopic techniques and are not amenable to quantification and kinetics analyses. As previosuly noted, quantification of the fibrils formed from soluble monomers, and perhaphs more importantly, the reverse process is important for advancing biomedical intervention. The screening of small-molecules that succesfully intervene in vitro in amyloid-forming trajectories are then translated into preclinical models.

Optical methods such as DLS or fluorescence using ThT or Congo red fluorophores to identify the presence of fibrils are often confounded by interference from small-molecule fluorescence. Others techniques such as solid-state NMR, are not amenable to easy use, lack access and fail to satisfactorily quantify the interconversion between the monomeric amyloid and intermediates and the mature fibril along the trajectory. The experimental sample preparation conditions do not recapitulate solution conditions.

Through several inroads, the method described here reduce barriers towards the study of amyloidogenesis which has traditionally involved elaborate sample preparation, mounting of “dried” samples, expensive instrumentation and protracted sample analyses times. Even though the technique is chemically and structurally “low-resolution” in nature, it provides a rapid, facile and inexpensive mechanism by which to quantify the loss of monomers (via their conversion to dimers, oligomers, proto-fibrils and fibrils). Importantly, by quantifying the intensity of the bands on the gel, it permits the user to build a kinetic profile of the consumption of monomers, formation of dimers, oligomers and finally the transformation of the amyloid protein into mature fibrils. From a biomedical perspective, the use of PAGE to establish a quantitative and dose-dependent profile of small-molecule efficiency in dissolving fibrils and oligomeric aggregates to their monomeric counterparts is highly desired.

## Conclusion

In conclusion, we demonstrate that a readily existing method and easily accessible appartatus can be used to obtain rich biophysical (kinetic) data about amyloid forming trajectories and the interplay between intermediates therein, with increased simplicity and reduced steps than current existing methods. Equally importantly, it can be used to screen small-molecules and also determine, via size analysis, where along the trajectory that the small-molecule intervenes. It increases accessibility to undergraduates, graduate students and advanced biomedical researchers in a variety of insitituions with a powerful, affordable, and simplified method, to study an important neurodegeneration-associated process.

## Figures and Tables

**Figure 4:**